# Development and testing the feasibility of a sports-based mental health promotion intervention in Nepal: a protocol for a pilot cluster-randomised controlled trial

**DOI:** 10.1186/s40814-023-01324-z

**Published:** 2023-08-24

**Authors:** Kelly Rose-Clarke, Damodar Rimal, Joanna Morrison, Indira Pradhan, John Hodsoll, Gerard Abou Jaoude, Brian Moore, Louise Banham, Justin Richards, Mark Jordans, Audrey Prost, Nabin Lamichhane, Jaya Regmee, Kamal Gautam, Nagendra P. Luitel

**Affiliations:** 1 Department of Global Health and Social Medicine, King’s College London, 40 Aldwych, London, WC2B 4BG UK; 2 Transcultural Psychosocial Organization Nepal, Baluwatar, , Kathmandu, Nepal; 3 Institute for Global Health, University College London, 30 Guilford Street, London, WC1N 1EH UK; 4 School of Teacher Education, Charles Sturt University, Panorama Avenue, Bathurst, NSW 2795 Australia; 5 School of Education and Social Sciences, University of the West of Scotland, Import Building, 2 Clove Cres, London E14 2B/ Foreign, Commonwealth and Development Office, King Charles St, London, SW1A 2AH UK; 6 Te Hau Kori, Faculty of Health, Victoria University of Wellington, PO Box 600, Wellington, 6140 New Zealand; 7 Institute of Psychiatry, Psychology and Neurosciences, King’s College London Centre for Global Mental Health, 16 De Crespigny Park, London, SE5 8AB UK

**Keywords:** Adolescent, Mental health promotion, Sport, Low- and middle-income countries, Community interventions, Nepal

## Abstract

**Background:**

Mental wellbeing encompasses life satisfaction, social connectedness, agency and resilience. In adolescence, mental wellbeing reduces sexual health risk behaviours, substance use and violence; improves educational outcomes; and protects mental health in adulthood. Mental health promotion seeks to improve mental wellbeing and can include activities to engage participants in sport. However, few high-quality trials of mental health promotion interventions have been conducted with adolescents, especially in low- and middle-income countries. We sought to address this gap by testing *SMART* (Sports-based Mental heAlth pRomotion for adolescenTs) in a pilot cluster-randomised controlled trial (cRCT) in Bardiya, Nepal.

**Methods:**

The objectives of the trial are to assess the acceptability and feasibility of SMART, test trial procedures, explore outcome distributions in intervention and control clusters and calculate the total annual cost of the intervention and unit cost per adolescent. The trial design is a parallel-group, two-arm superiority pilot cRCT with a 1:1 allocation ratio and two cross-sectional census surveys with adolescents aged 12–19, one pre-intervention (baseline) and one post-intervention (endline). The study area is four communities of approximately 1000 population (166 adolescents per community). Each community represents one cluster. SMART comprises twice weekly football, martial arts and dance coaching, open to all adolescents in the community, led by local sports coaches who have received psychosocial training. Sports melas (festivals) and theatre performances will raise community awareness about SMART, mental health and the benefits of sport. Adolescents in control clusters will participate in sport as usual. In baseline and endline surveys, we will measure mental wellbeing, self-esteem, self-efficacy, emotion regulation, social support, depression, anxiety and functional impairment. Using observation checklists, unstructured observation and attendance registers from coaching sessions, and minutes of meetings between coaches and supervisors, we will assess intervention fidelity, exposure and reach. In focus group discussions and interviews with coaches, teachers, caregivers and adolescents, we will explore intervention acceptability and mechanisms of change. Intervention costs will be captured from monthly project accounts, timesheets and discussions with staff members.

**Discussion:**

Findings will identify elements of the intervention and trial procedures requiring revision prior to a full cRCT to evaluate the effectiveness of SMART.

**Trial registration:**

ISRCTN, ISRCTN15973986, registered on 6 September 2022; ClinicalTrials.gov, NCT05394311, registered 27 May 2022.

## Background

Defined as more than the absence of mental health problems, mental wellbeing is a positive mental state that enables individuals to thrive [1]. Mental wellbeing encompasses life satisfaction, social connectedness, agency and resilience and is associated with improved physical health including reduced mortality, better immune responses and cardiovascular health [1–5]. In adolescence, mental wellbeing, especially social connectedness and agency, reduces sexual health risk behaviours, substance use and violence; improves educational outcomes; and protects mental health in adulthood [6, 7].

Mental wellbeing is a target for mental health promotion interventions which seek to empower people through policies, social and economic interventions and programmes that facilitate healthier lives [8]. There is a lack of high-quality trials and hence evidence for these interventions, especially in low- and middle-income countries (LMICs) where around 90% of the world’s adolescents live [9]. Consequently, the 2018 Lancet Commission for Global Mental Health called for more focus on mental health promotion research [10].

The World Health Organization (WHO) and United Nations Children’s Fund (UNICEF) Helping Adolescents Thrive (HAT) Toolkit outlines possible approaches to mental health promotion among adolescents [11, 12]. One of these approaches is participation in sport, which is promising for three main reasons. First, participation in sport during adolescence can reduce depression and anxiety symptoms, loneliness and stress [13]. Second, sport may promote mental health and wellbeing through neurobiological, behavioural and psychosocial mechanisms. The strongest evidence is for psychosocial mechanisms, through improvements in self-efficacy, self-esteem and emotion regulation [14, 15]. Third, sport could be an equitable platform for intervention because it can engage younger and older adolescents, in and out of school, across genders and socioeconomic backgrounds.

The evidence base for sports-based mental health promotion is only just emerging [16]. In low- and middle-income countries, there have been few studies and results have been mixed. In Pakistan, an intervention using sports and games to build life skills reduced depression among 6th and 8th grade public school students (approx. aged 12–14) [17]. In Uganda, an 11-week competitive football league increased depression and anxiety-like symptoms among boys and there was no effect on girls [18]. These findings suggest there are contextual factors related to the delivery of sport, the local community and the programme setting that influence how sports impact mental health and wellbeing. Formative work and piloting are crucial to understand community barriers and facilitators, identify suitable facilitators and venues, explore adolescents’ priorities and preferences for intervention and identify how to promote the positive aspects of sports.

We sought to fill the evidence gap for sports-based mental health promotion in LMICs by developing and testing *SMART* (Sports-based Mental heAlth pRomotion for adolescenTs) to improve mental wellbeing among adolescents aged 12–19 in Nepal. We focus on Nepal because adolescents in this setting are exposed to many risk factors for mental health problems (e.g. poverty, corporal punishment, gender-based violence, effects of climate change and earthquakes, inter-caste/ethnicity tensions), and beyond family and friends there is limited mental health support. SMART involves training local sports coaches and assistant coaches in psychosocial skills. It was informed by (i) a qualitative study with adolescents, parents, teachers and coaches to identify community needs, preferences and priorities for adolescent mental health promotion and participation in sport; (ii) mapping sports for development programmes in Nepal; and (iii) a global review of randomised controlled trials of sports-based interventions for adolescent mental health to consolidate local and global learning on sports-based programming. The intervention development process and theory of change will be described elsewhere. This paper describes the SMART pilot trial protocol.

### Aim and objectives

We aim to evaluate the feasibility and acceptability of sports-based mental health promotion by coaches and trial procedures in a rural Nepali setting. The objectives are to:

1. Assess the feasibility and acceptability of SMART
2. Test trial procedures including cluster randomisation, blinding, outcome measures and safety standard operating procedures
3. Explore outcome distributions in intervention and control clusters
4. Calculate the total annual cost of the intervention and unit cost per adolescent participant, compared with the control, from a provider perspective

## Study design and methods

### Setting

Transcultural Psychosocial Organization (TPO) Nepal (www.tponepal.org) is leading the study with King’s College London (KCL). The study is in Bardiya district, in Lumbini Province, on the Indian border. In 2021, the estimated district population was 459,900 [19]. More than 50% are of Tharu ethnicity, indigenous to this area of Nepal, and more than half speak Tharu as their first language [20]. Eighty-three percent of men and 72% of women aged five and over can read and write [19]. The district has one hospital, three primary health centres and 30 health posts [21]. The most common sport in Bardiya is football but cricket, volleyball and *kabbadi* (a contact team sport popular across South Asia) are also played. Adolescents mostly engage in sport through school. Community football clubs exist but are mainly for boys. There are few opportunities for girls to play sport in the community.

Bardiya is administratively divided into eight municipalities. Each municipality is comprised of multiple wards. The study area is four communities of approximately 1000 population, the size of a small ward, in one of these municipalities. Each community represents one cluster. Communities will be selected on the basis that they are far enough apart and/or separated by natural boundaries (e.g. jungle or farmland) so that it is unlikely adolescents from the control communities will participate in activities in intervention communities; they are accessible to the study team by road; and they are ethnically diverse (and hence findings will be more generalisable to other areas of Nepal).

### Design

We are doing a parallel-group, two-arm superiority pilot cluster-randomised controlled trial (cRCT) with a 1:1 allocation ratio and two cross-sectional surveys, one pre-intervention (baseline) and one post-intervention (endline). Trials using this design have been used to evaluate community-based interventions including women’s groups in Nepal, India and Bangladesh [22–24] and adolescent groups in India [25]. In a fully powered trial using this design, the primary analysis would be a cross-sectional comparison across trial arms of outcomes from the endline survey, adjusted for baseline differences. However, because our trial is an underpowered pilot trial, we will not be testing between-group differences.

We chose this trial design because a cross-sectional comparison of trial arms rather than a longitudinal approach is advantageous. Adolescents are highly mobile and we anticipate a substantial proportion will move in or out of the study clusters for employment, marriage, or education and we would not be able to follow them up. We chose a design which enables us to examine the effects of the intervention at the community level because (i) emerging evidence emphasises the importance of community social cohesion in protecting adolescent mental health [26] and the newly published WHO-UNICEF HAT guidelines recommend community-based universal approaches to adolescent mental health promotion [12]; (ii) there are likely to be benefits of a sports-based intervention that extend beyond the participants themselves to their supporters and peer network, including enhanced social interaction and connectedness; and (iii) an open intervention model is more inclusive and therefore ethical, and in line with existing community sports programmes in this setting.

### Trial participants and target population

For the baseline and endline surveys, we aim to recruit all eligible adolescents aged 12 to 19 living in the four study clusters. Although the WHO defines adolescence as the age range from 10 to 19 [27], we will not include adolescents aged 10 and 11 because findings from our formative qualitative research suggest adolescents attending secondary school (aged 12 to 19) do not want to participate in sports coaching with those attending primary school. We will exclude adolescents who do not permanently reside in the study clusters (i.e. adolescents who live outside the clusters but are visiting friends/family in the study area at the time of the survey). Intervention activities are open to any adolescents aged 12–19 who wish to participate, but the target population will be those living in the two intervention study clusters.

### SMART intervention

**Fig. 1:**
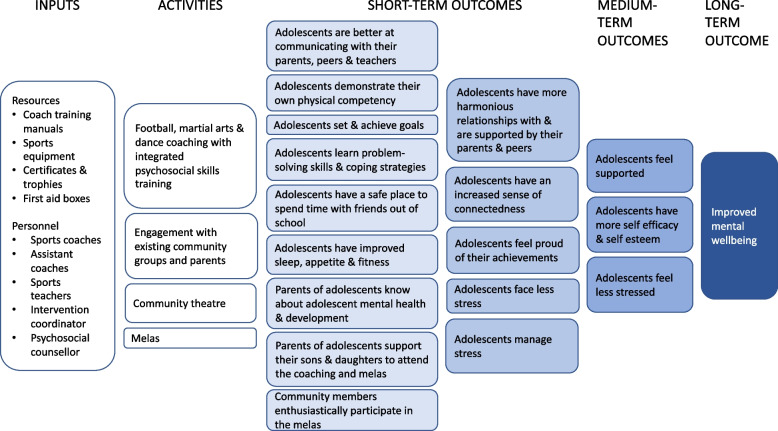
Overview of the SMART intervention

Figure 1 provides an overview of the intervention which aims to improve adolescent mental wellbeing through sports coaching with integrated psychosocial skills training. In each intervention cluster, we will offer coaching in football, dance and martial arts. We chose these sports because adolescents participating in our formative research said they were interested to receive coaching in these sports and because the coaches were available in Bardiya.

Adolescents can participate in one or more sports at the same time, attending as many or as few coaching sessions as they wish. Coaching for each sport will be implemented in parallel over 10 months. Coaching sessions will be 90 min and held twice per week on school premises or community sports grounds. Each session will include training on sports and psychosocial skills including interpersonal communication, self-awareness, assertiveness, coping and problem-solving. Adolescents will work on these skills through drills, games, role-play, discussions and relaxation exercises. They will be encouraged to apply the skills outside coaching sessions, in school and at home.

For each sport, sessions will be led by a locally recruited sports coach and an assistant coach. Coaches will be recruited through open advertisement and personal networks. Eligibility criteria include experience coaching in martial arts, dance or football and the ability to live in the study area for the duration of the intervention. Coaches will receive an 11-day training programme led by psychosocial counsellors covering concepts of adolescent mental health, orientation to SMART and the role of a SMART coach, foundational helping skills (empathy, verbal and non-verbal communication, listening, etc.), group facilitation skills, safeguarding, self-care and basic first aid. Coaches will practice sessions with small groups of adolescents and receive feedback from counsellors.

Through our formative research, we learned about some of the barriers to adolescents playing sport, especially among girls. These include girls feeling uncomfortable playing with boys and a male coach, feeling vulnerable and at risk of sexual assault and lack of knowledge about the rules of the sport. We will try to address these barriers in the intervention by (i) recruiting a female coach or assistant coach for each sport, (ii) running some sessions on school sports grounds where girls feel safe and do not have to travel far from their homes and (iii) assuming no previous knowledge or experience of the rules and providing an orientation in coaching sessions.

Coaches will organise sports *melas* (festivals) at the start, midway and at the end of the 10-month implementation period. Melas will involve contributions from football, martial arts and dance, including football matches, martial arts demonstrations and dance performances, and will serve as a platform for adolescents to try out different sports and engage with their community about adolescent mental health and the benefits of sports for mental wellbeing. Sports coaches and assistant coaches will meet with existing community groups (mothers’ groups, micro-credit groups, parent/teacher committee) to explain the intervention and its potential benefits for adolescents. Coaches will support individual adolescents to attend coaching sessions, for example, by meeting with adolescents and their parents. We will also work with a theatre group to create short, community dramas to promote adolescent mental health and participation in the intervention. A detailed description of the intervention theory of change is the focus of a forthcoming publication.

### Control: enhanced usual care

We will distribute sports equipment (e.g. footballs, nets, kit) at the municipality level and adolescents in control clusters will be free to participate in sports as usual.

Most people with mental health problems in Bardiya do not receive psychological or psychiatric intervention due to the lack of local mental health services. We will conduct training for health workers in the municipality based on the WHO mental health Gap Action Programme Humanitarian Intervention Guide which has been contextualised for Nepal [28]. In both control and intervention clusters, we will provide information about how to access these trained health workers. Adolescents identified as being at high risk of suicide will be referred to a psychosocial counsellor employed through the project.

### Recruitment

Trained researchers will recruit participants for the cross-sectional surveys by going house to house in all four study clusters, asking members of each household if there are any adolescents living there.

To recruit participants for the intervention activities, we will publicise information about coaching through schools, community theatre performances and at sports melas in the intervention clusters.

### Outcomes and tools

To assess the primary outcome, mental wellbeing, we will use the Warwick Edinburgh Mental Wellbeing Scale [29]. For secondary outcomes, we will use the Rosenberg Self-Esteem Scale [30], Schwarzer Self-Efficacy Scale [31], Adolescent Emotion Regulation Strategies Questionnaire [32] and Multidimensional Scale of Perceived Social Support [33] to measure self-esteem, self-efficacy, emotion regulation and social support respectively. Tools to measure primary and secondary outcomes have been translated and adapted for Nepal and will be validated using data from the trial baseline survey. We will also measure depression using the Patient Health Questionnaire 9 items—modified for adolescents (PHQ-A) and the Generalised Anxiety Disorder Assessment (GAD-7) to measure anxiety [34, 35]. Functional impairment will be assessed using a tool developed by the research team which asks about 11 locally relevant, age-appropriate daily activities (e.g. working in the fields, doing household chores and doing homework) and, in the past 2 weeks, if there was any difficulty completing them. Response options are 0 “None of the time”, 1 “A little of the time”, 2 “Some of the time” and 3 “Most of the time”. Higher scores indicate more functional impairment. Measures are described in Table 1.

**Table 1:** Trial outcomes and tools

Outcome	Tool	No. items	Score range
**Primary outcome**			
Mental wellbeing	Warwick Edinburgh Mental Wellbeing Scale	14	14–70
**Secondary outcomes**			
Self-efficacy	Schwarzer Self-Efficacy Scale	10	10–40
Self-esteem	Rosenberg Self-Esteem Scale	10	10–40
Emotion regulation	Adolescent Emotion Regulation Strategies Questionnaire	23	0–92
Social support	Multidimensional Scale of Perceived Social Support	12	12–60
**Other outcomes**			
Depression	Patient Health Questionnaire 9-item—modified for adolescents	9	0–27
Anxiety	Generalised Anxiety Disorder 7-item	7	0–21
Functional impairment	Locally developed tool	11	0–33

### Randomisation

An independent researcher who is not familiar with the study setting will randomise clusters before the baseline survey. Clusters will be paired and one cluster in each pair will be randomised to the intervention arm and one to control. One pair will be semi-urban clusters near the main highway. The other pair will be rural clusters far from the road and relatively inaccessible. The researcher will use SPSS software to do the randomisation.

### Blinding and concealment

The research and intervention teams will work independently from separate offices. Researchers conducting the baseline and endline surveys will not be given information about the allocation of clusters but may encounter some of the intervention activities and participants. Due to the participatory nature of the intervention, adolescents, sports coaches and assistant coaches will not be blind to allocation. The researcher conducting the final pilot trial analysis will be blind to allocation.

### Sample size

The decision to include four clusters was informed by the available budget and resources. Four clusters will enable us to pilot intervention implementation in two sites and test the randomisation technique. In Nepal, 11.2% of adolescents are aged 15–19; 10.4% are 10–14 [36]. Around 6.2% will therefore be aged 12–14. In each cluster of c.1000 population, we expect there will be around 166 adolescents aged 12–19—i.e. 17.4% of the population. In total, in the four clusters, we expect there to be 696 adolescents. Some adolescents will be living away from home for work or study. Assuming 20% are living away, we will sample 556 adolescents. Although we will estimate the intra-cluster correlation coefficient and recruitment rate using the baseline data, these will be biased downwards due to the small number of clusters [37]. In calculating the sample size for a future full-scale cRCT, we will triangulate our estimates with those from the wider literature including other studies in Nepal and trials of mental health promotion interventions to ensure the trial is fully powered.

### Data collection, management and monitoring

Researchers will find a convenient time to conduct the interview with the adolescent in their home or another private, convenient place. If an adolescent is not available when the researcher visits, the reason will be recorded. The researcher will try up to three times to conduct the interview. In interviews, we will collect data on adolescent mental health and wellbeing, household socioeconomic status, sports preferences and participation in the intervention activities. The survey will take around one hour to complete. We will use the KoboToolbox data collection platform. Researchers will enter data on mobile phones or tablets. We will use automated skip patterns and consistency logic to reduce errors and missing data.

During the surveys, the project coordinator will regularly download data from the server to check the number of interviews completed and identify any problems such as outliers, errors or missing data. We will pseudonymise the final dataset and store it on TPO Nepal’s secure central server and KCL Sharepoint.

### Process evaluation

We will explore how the intervention is implemented, mechanisms through which it may bring about change, and contextual factors that might affect outcomes and implementation. In intervention clusters, we will collect data on intervention fidelity; exposure and reach using observation checklists; unstructured observation of coaching sessions; minutes of meetings between coaches, assistant coaches and the intervention supervisors; and attendance registers from coaching sessions. We will also conduct focus group discussions and interviews with key stakeholders (i.e. coaches, teachers, caregivers and adolescents) at the end of the intervention. Table 2 lists the process evaluation data collection methods. We will analyse quantitative data from the baseline and endline survey. Interviews and focus groups will last around one hour. Adolescents will be recruited at intervention activities (intervention participants) or in schools (non-participants). Caregivers will be recruited through coaches and teachers. Key stakeholders will be approached directly by the research team.

**Table 2:** Process evaluation data collection

Method	Sample	Process indicator
Quantitative data collected by coaches on the number of returning adolescents, number of new adolescents, their approximate age, gender and ethnicity at each session	All	Reach
Quantitative data collected using a checklist by intervention coordinator on key intervention features	All	Fidelity
Unstructured observation by intervention coordinator—field notes about coaching sessions and informal conversations	7 observations per sport per 14-week intervention block	Fidelity, context, mechanism
Meeting minutes from community advisory groups	All	Context, mechanism
Meeting minutes from monthly meetings between intervention coordinator, coaches and assistant coaches	All	Fidelity, context, mechanism
Focus group discussions with boys who participated in the intervention (age 12–15)	1 endline (2 clusters)	Context, mechanism
Focus group discussions with boys who participated in the intervention (16–19)	1 endline	Context, mechanism
Focus group discussion with boys that didn’t participate (sampled based on consultation with sports coaches)	1 endline	Context, mechanism
Focus group discussion with girls who participated in the intervention (12–15)	1 endline	Context, mechanism
Focus group discussion with girls who participated in the intervention (16–19)	1 endline	Context, mechanism
Focus group discussion with girls that didn’t participate (sampled based on consultation with sports coaches)	1 endline	Context, mechanism
Group interviews with coaches (*n* = 3)	1 midline 1 endline	Fidelity, context, mechanism
Focus group discussion with assistant coaches (*n* = 6)	1 midline 1 endline	Fidelity, context, mechanism
Group discussion or group interview with female caregivers (one in each cluster)	2 endline	Context, mechanism
Group discussion on group interview with male caregivers (one in each cluster)	2 endline	Context, mechanism
Interview with teachers	1 per school	Fidelity, context, mechanism

Adolescents will be purposively sampled based on their gender, age and participation (or not) in the intervention. Caregivers will be purposively sampled based on the gender and age of their child—we would like a mix of genders and ages of adolescents. Teachers will be purposively sampled as the key point person in the school for the intervention. All sports coaches and assistant coaches will be sampled. Sampling will also be informed by any differences that we observe whilst implementing the intervention.

In addition to the process evaluation, we will meet with three community advisory groups of (i) adolescents, (ii) caregivers and (iii) teachers and health workers who are living or working in the intervention clusters. We will ask the groups for their perspectives on the intervention, including any unexpected harms or benefits. Information from these meetings will inform the content of ongoing training and supervision of the coaches, logistics and adolescent recruitment strategies and contribute to the process evaluation. Each group will have six to eight members, recruited from the intervention clusters, representing local diversity in terms of caste/ethnic group and gender. We will sample younger and older adolescents and the caregivers of younger and older adolescents. Groups will meet twice: after the first 14-week block of sessions and mela, and at the end of the intervention.

### Participant incentives

We will cover transportation costs incurred by adolescents and adults participating in the process evaluation and community advisory groups. They will also be offered refreshments (juice and biscuits). No incentives will be offered to adolescents participating in the baseline or endline survey, or coaching sessions.

### Post-trial care

Where required, a psychosocial counsellor employed by TPO Nepal will remain in the study setting to provide follow-up care for study participants.

### Ethics and research governance

The trial has ethical approval from King’s College London (RESCM-22/23–27,152; HR/DP-22/23–33,427) and the Nepal Health Research Council (NHRC) Ethics Committees (Ref no. 3088).

The NHRC requires caregiver consent and adolescent assent for adolescents under the age of 18, which we will adhere to in this study. We will collect consent for participation in the baseline survey and again for the endline survey. A trained researcher will explain the study to the adolescent and caregiver and give them a consent/assent form and information sheet (in Nepali). The researcher will ask the adolescent and caregiver if they have any questions about the study and make sure they are satisfied before asking them both to sign the consent/assent form. We will stress that just because the adolescent participated in the baseline survey and/or the intervention activities they are not obliged to participate in the endline survey. We will also ask for consent/assent from adolescents participating in the coaching sessions and consent from their caregiver where necessary. Sports coaches will collect the signed consent/assent forms at the start of the sessions. For the process evaluation and community advisory groups, participants (adults and adolescents) interested in participating will be given a consent/assent form and information sheet. Participants (and caregivers) will be able to ask researchers any questions about the study either in person or by phone (contact details on the information sheet). Those who return signed consent/assent forms will be able to participate.

### Harms

There is no data safety monitoring committee for this trial: adverse events and severe adverse events will be reported to an independent trial steering committee. Participant information sheets will outline the research topic (mental health promotion) and, where applicable, participants will be asked about mental health problems. Researchers will be trained to ask questions in a sensitive and culturally respectful way and to conduct interviews/focus group discussions in a private space where participants feel comfortable. Participants will be reminded that they can stop or pause if they are uncomfortable and that they do not have to answer questions if for any reason they do not want to. If participants do become visibly distressed, researchers will ask if they would like to take a break or finish and will follow up with them individually. If participants disclose suicidal behaviour, the researcher will activate a standard operating procedure which involves arranging an urgent assessment by the TPO Nepal psychosocial counsellor who is based in the study area. Cases will be documented and submitted to the project coordinator and principal investigators for review.

Adolescents may disclose mental health problems and abuse to a coach or assistant coach during the intervention. The psychosocial counsellor will assess these cases and formulate a plan which may involve local health services and services for survivors’ support.

If adolescents incur an injury due to the intervention (e.g. during the sports coaching), coaches and/or assistant coaches will assess the severity, consult with the adolescent and their caregiver and help them to access appropriate health facilities if necessary. Coaches will have access to a basic first aid kit.

## Planned analyses

### Qualitative data

Audio recordings of qualitative interviews and focus group discussions will be transcribed in Nepali or Tharu and translated into English. We will analyse English transcripts along with observation notes using the Framework Method [38]. We will develop an analytical framework informed by the intervention theory of change and including deductive codes related to feasibility and acceptability. Several members of the research team will be involved in coding transcripts. All coders will initially code the same three transcripts and compare coding to check consistency.

We will take notes during meetings with community advisory groups which will inform the ongoing intervention process and process evaluation tools.

### Quantitative data

JH, NPL, KRC and DR will have access to the final trial dataset. JH will conduct the main analyses. Quantitative outcomes will be descriptively summarised (mean and standard deviation for continuous data or counts and proportions for categorical) for each trial arm at baseline and endline. We will obtain preliminary estimates of differences in primary and secondary outcomes by trial arm at the cluster level. By linking baseline and endline data at an individual level, we will explore differences for adolescents who participated in intervention activities on an intention to treat and per protocol basis. Although the number of clusters is small, the anticipated sample size per cluster should be sufficient to assess factors predictive of participant attrition and adherence (for example due to person mobility) [39]. Individual outcomes (and in a full trial—cluster-level outcomes) can be evaluated taking into account pre-intervention and post-intervention predictors of missingness and adherence using recent developments leveraging machine learning in causal estimators such as targeted maximum likelihood estimation [40].

### Cost analysis

We will calculate the costs of setting up and implementing the intervention, compared with the control, from the provider perspective. Costs from monthly project accounts will be entered into an Excel costing tool, categorised as start-up or implementation costs and allocated to different cost centres (capital, staff and materials) and activities such as sports coaching or monitoring and evaluation. Costs related to research activities will be captured but excluded from the results. Project staff timesheets will inform how much of staff salaries should be allocated to set-up and implementation and across different activities. Staff will be asked about their time allocations at the end of manual development, ahead of implementation and at the end of implementation. Both financial and economic costs will be calculated. Indirect costs that are not captured by project accounts such as the opportunity cost of volunteer time or donated goods will be identified through project records and discussions with staff members and then assigned a value using local secondary data or assumptions. Results will include the total annual cost of SMART compared with the control, annual implementation costs, unit cost per adolescent and cost per cluster. One and two-way sensitivity analyses will be carried out by varying key inputs with uncertainty such as joint costs and staff time allocations.

### Mixed methods framework

Overall, we will use a fully mixed concurrent equal status mixed methods design that mixes qualitative and quantitative research across the research objectives, type of data and data inference [41]. In the process evaluation, quantitative findings relating to reach and fidelity will be explored through qualitative research, using a fully mixed sequential equal status design. We will use the Good Reporting of A Mixed Methods Study (GRAMMS) Framework to inform and describe the collection, analysis, integration and interpretation of quantitative and qualitative data [42].

## Progression criteria

Related to the study objectives, we have identified criteria to inform the decision to progress to a fully powered cRCT:

- Fidelity to the intervention manual—at least 75% of activities in each session completed at a satisfactory/superior level according to a fidelity checklist developed for the intervention.
- Exposure to the intervention—at least 30% of adolescents aged 12–19 living in the intervention clusters participate in at least five coaching sessions, measured in the endline survey. This is based on our assumption that 30% of adolescents need to participate to see effects at the community level and five sessions are sufficient for adolescents to build their psychosocial skills.
- Fewer than 10% of adolescents experience an adverse event (i.e. an untoward physical or psychological occurrence which is potentially related to the intervention) or a severe adverse event (i.e. an adverse event that is life-threatening, results in death and/or requires hospitalisation).
- Fewer than 15% missing items on mental health and wellbeing outcome measures at endline
- Fewer than 10% of adolescents living in the control clusters participated in the intervention.

Where criteria are not met, we will revisit the intervention/trial procedure in depth before progressing to a full trial.

## Dissemination

We will publish findings from the trial in academic journals. We will organise dissemination events in Bardiya and Kathmandu, with national and local government officials, adolescents, caregivers, teachers, researchers and non-governmental organisations.

## Timeline

Recruitment and data collection for the baseline survey and facilitator training started in Jan 2023. Table 3 presents the SPIRIT (Standard Protocol Items: Recommendations for Interventional Trials) enrolment, interventions and assessment schedule.

**Table 3:** SPIRIT schedule of enrolment, interventions, and assessments for SMART

	**Enrolment**	**Allocation**	**Baseline**	**Intervention implementation**	**Endline**
**Timepoint**	***-t***_***1***_		**t**_**0**_		***t***_***1***_
**Enrolment:**					
Identification of study clusters	X				
Cluster randomisation and allocation		X			
Adolescent eligibility screen			X		
Participant informed consent			X		
**Intervention:**					
*SMART*				X	
*Control*				X	
**Assessments:**					
*Warwick Edinburgh Mental Wellbeing Scale*			X		X
*Schwarzer Self-Efficacy Scale*			X		X
*Rosenberg Self-Esteem Scale*			X		X
*Adolescent Emotion Regulation Strategies Questionnaire*			X		X
*Multidimensional Scale of Perceived Social Support*			X		X
*Patient Health Questionnaire 9-item—modified for adolescents*			X		X
*Generalised Anxiety Disorder 7-item*			X		X
*Functional impairment tool*			X		X

## Discussion

We have developed a community sports intervention for adolescents in Nepal, which will help to address the scarcity of evidence-based mental health promotion interventions for children and adolescents in LMICs. Strengths of our study design include (i) an intervention informed by evidence at the local and global level working with locally available resources and personnel; (ii) a relatively long intervention period to maximise any benefits; (iii) a pragmatic cross-sectional evaluation approach to account for the mobility of adolescents, and to explore any effects of sport for those not directly participating in the intervention activities; and (iv) community advisory groups and a process evaluation to provide insight into how the intervention is being implemented and may effect change. Limitations of the study include the lack of blinding among coaches, assistant coaches, teachers and intervention supervisors, and the small number of clusters and potential baseline differences between control and intervention arms. Findings from the study will inform the decision to progress to a fully powered cluster-randomised controlled trial. Where findings point to feasibility and acceptability issues, for example in terms of randomisation, fidelity to the intervention manual, and safety procedures, these will be addressed and modified in the full trial protocol.

### Trial status

The trial is open and recruiting participants for the baseline survey. Any amendments to this protocol will be communicated to the Research Ethics Committees and trial steering committee.

## Data Availability

Data sharing is not applicable to this article as no datasets were generated or analysed during the current study.
