# Correction: Development and testing the feasibility of a sports‑based mental health promotion intervention in Nepal: a protocol for a pilot cluster‑randomised controlled trial

**DOI:** 10.1186/s40814-024-01451-1

**Published:** 2024-01-29

**Authors:** Kelly Rose‑Clarke, Damodar Rimal, Joanna Morrison, Indira Pradhan, John Hodsoll, Gerard Abou Jaoude, Brian Moore, Louise Banham, Justin Richards, Mark Jordans, Audrey Prost, Nabin Lamichhane, Jaya Regmee, Kamal Gautam, Nagendra P. Luitel

**Affiliations:** 1 Department of Global Health and Social Medicine, King’s College London, 40 Aldwych, London, WC2B 4BG UK; 2 Transcultural Psychosocial Organization Nepal, Baluwatar, Kathmandu, Nepal; 3 Institute for Global Health, University College London, 30 Guilford Street, London, WC1N 1EH UK; 4 School of Teacher Education, Charles Sturt University, Panorama Avenue, Bathurst, NSW 2795 Australia; 5 School of Education and Social Sciences, University of the West of Scotland, Import Building, 2 Clove Cres, London E14 2B/ Foreign, Commonwealth and Development Office, King Charles St, London, SW1A 2AH UK; 6 Te Hau Kori, Faculty of Health, Victoria University of Wellington, PO Box 600, Wellington, 6140 New Zealand; 7 Institute of Psychiatry, Psychology and Neurosciences, King’s College London Centre for Global Mental Health, 16 De Crespigny Park, London, SE5 8AB UK

**Correction: Pilot Feasibility Stud 9, 149 (2023)**

**https://doi.org/10.1186/s40814-023-01324-z**

Following publication of the original article [1], the authors wish to update the author list to include John Hodsoll, identified as JH in the original article.

The original article [1] has been updated.
